# Improving the Accuracy in Classification of Blood Pressure from Photoplethysmography Using Continuous Wavelet Transform and Deep Learning

**DOI:** 10.1155/2021/9938584

**Published:** 2021-08-05

**Authors:** Jiaze Wu, Hao Liang, Changsong Ding, Xindi Huang, Jianhua Huang, Qinghua Peng

**Affiliations:** ^1^Institute of TCM Diagnostics, Hunan University of Chinese Medicine, Changsha, Hunan 410208, China; ^2^Post-Doctoral Research Station of Integrative Medicine, Hunan University of Chinese Medicine, Changsha, Hunan 410208, China; ^3^School of Informatics and Engineering, Hunan University of Chinese Medicine, Changsha, Hunan 410208, China; ^4^Institute of Herbs, Hunan Academy of Chinese Medicine, Changsha, Hunan 410208, China

## Abstract

**Background:**

Continuous wavelet transform (CWT) based scalogram can be used for photoplethysmography (PPG) signal transformation to classify blood pressure (BP) with deep learning. We aimed to investigate the determinants that can improve the accuracy of BP classification based on PPG and deep learning and establish a better algorithm for the prediction.

**Methods:**

The dataset from PhysioNet was accessed to extract raw PPG signals for testing and its corresponding BPs as category labels. The BP category of normal or abnormal followed the criteria of the 2017 American College of Cardiology/American Heart Association (ACC/AHA) Hypertension Guidelines. The PPG signals were transformed into 224 *∗* 224 *∗* 3-pixel scalogram via different CWTs and segment units. All of them are fed into different convolutional neural networks (CNN) for training and validation. The receiver-operating characteristic and loss and accuracy curves were used to evaluate and compare the performance of different methods.

**Results:**

Both wavelet type and segment length could affect the accuracy, and Cgau1 wavelet and segment-300 revealed the best performance (accuracy 90%) without obvious overfitting. This method performed better than previously reported MATLAB Morse wavelet transformed scalogram on both of our proposed CNN and CNN-GoogLeNet.

**Conclusions:**

We have established a new algorithm with high accuracy to predict BP classification from PPG via matching of CWT type and segment length, which is a promising solution for rapid prediction of BP classification from real-time processing of PPG signal on a wearable device.

## 1. Introduction

Elevated blood pressure (BP) has been a potent issue inducing stroke, heart attack, and kidney failure. Though some proven and well-tolerated lifestyle and drug treatment strategies can control BP, it remains the major preventable cause of cardiovascular diseases (CVD) and all-cause death in the world [1]. Accurate BP measurement and recording are essential to grade BP level, ascertain BP-related CVD stratification, and guide the management of hypertension. Cuff-based BP measurement devices have been still widely used in hospital settings to detect abnormal BP. However, in patients treated for hypertension, both “white coat effect” (higher office BPs than out-of-office BPs) and “masked uncontrolled hypertension” (controlled office BPs but uncontrolled BPs in out-of-office settings) are difficult to detect [2]. ABPM and HBPM help doctors follow the risk profiles of the patients' white coat hypertension and masked hypertension counterparts, respectively [3, 4]. Both ABPM and HBPM typically based on multiple measurements of BP provide better methods to predict long-term CVD outcomes than office BP [4].

Office BP measurement in hospital, ABPM, and HBPM often require the technique by pressurizing and releasing an upper arm cuff to detect systolic and diastolic pressure. This old method is inconvenient and difficult to monitor BP persistently due to physiological limitations, for example, periodic cuff inflation and deflation will disturb sleeping to prevent the real BP detection during sleep. Some new methods of cuff-less BP detection and evaluation have been proposed, such as estimation of BP and using photoplethysmography (PPG) and electrocardiogram (ECG) signals. Although the pulse transit time (PTT) or pulse arrival time (PAT) method extracted from PPG and ECG achieved overall acceptable accuracy for BP estimation [5, 6], the algorithm required at least two signals, increasing the operational complexity and cost for wearable devices. PTT must deal with different physiological parameters, so a calibration procedure is required [7]. Synchronization is another issue in using these signals in real-time because when recording two or more signal channels via independent systems, an uncontrollable artificial delay exists as the data acquisition is started manually [8]. The former study even reported that PTT has a negative correlation with systolic BP, which is not reliable enough to become a surrogate marker of systolic BP [9]. It is clinically and practically desirable to apply the PPG signal only for estimating BP. Riaz et al. reported that the simplest linear classifiers produce satisfying results for indicating classes of normal or abnormal BP according to the proposed PPG wave feature [10]. Khalid et al. compared three machine learning algorithms (regression tree, multiple linear regression, and support vector machine) via pulse features to estimate BPs using raw PPG signals and concluded that the regression tree algorithm was the best approach. PPG wave can be transformed into scalograms by fast Fourier transform (FFT) or continuous wavelet transform (CWT). The transformations promote recognition and learning by computer. FFT naturally applying to stationary signals whose frequency contents do not change is unable to tell when these frequency components exist in time [11], but almost all biological signals are nonstationary. CWT maps the time and frequency information simultaneously by the time-frequency plane and helps to investigate what spectral components exist at any given interval of time from biological signals, such as ECG and PPG. Mansouri SR et al. employed a deep convolutional neural network (CNN) to estimate BP through the scalogram representing a CWT transformed from PPG and ECG waves and demonstrated a low root mean square error (RMSE) rate of 3.36 mmHg and high accuracy of 86.3% [12].

Previous studies usually focused on one factor individually, such as signal transform feature extraction, or new deep learning method, but CWT waveform, segment length, and methods are common key factors for accuracy of prediction. CWTs generate distinct scalograms according to different classes of wavelet bases (Shannon, Mexican hat, Morlet, etc.) and segment length, but the recent studies used the same scalogram (MATLAB default/Morse wavelet transform) with a fixed length of PPG segment (5 s) for BP classification by deep learning [12, 13]. Whether CWT type and segment length could influence the accuracy of the classification is still unknown, and the optimal combination based on these factors also need to be established. This research aimed to find out the determinants to improve the accuracy of BP classification using PPG and deep learning and establish a better algorithm for BP prediction.

## 2. Materials and Methods

### 2.1. Dataset

The data were from Multiparameter Intelligent Monitoring in Intensive Care-III (MIMIC-III) Waveform Database provided by PhysioNet [14]. In brief, the database contains 67,830 record sets for approximately 30,000 intensive care unit (ICU) patients, and almost all record sets include a waveform record containing signals of continuous arterial blood pressure (ABP) waveforms, fingertip PPG signals, ECG, and respiration. The data with patient ID and corresponding available PPG and ABP signals were extracted for analysis in our study. A manual check was conducted to exclude the records with the movement artefact, missing peaks, no signal, and so on. Finally, 311,000 signals with a frequency of 125 Hz were put into the analysis, and the first 90% of the randomized data from the dataset are used for training and the rest are for testing.

### 2.2. Hypertension Criteria

The ABP signals were used to label the blood pressure levels and confirm the classification of BP. The records were divided into normal or abnormal following the 2017 American College of Cardiology/American Heart Association (ACC/AHA) Hypertension Guidelines [3].

### 2.3. Signal Preprocessing

The ABP and PPG signals were used as the target source and predicted source, respectively. Each PPG segment was firstly processed with a moving average filter by Python function (numpy. convolve). This filter is a moving average filter to smooth the PPG signal. Baseline wandering caused by the respiratory activity was also removed from the segments. In a segment, the mean value of synchronous ABP wave peaks was calculated as systolic pressure and the mean value of wave troughs as diastolic pressure. Then, the BP category was labelled as described in the ACC/AHA Hypertension Guidelines.

### 2.4. PPG Signal Transformation

**Figure 1:**
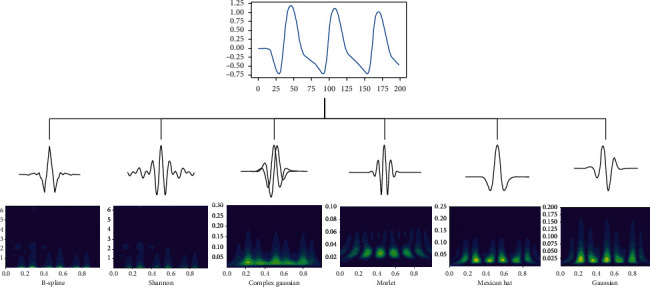
The transformed colorful 2D scalograms by different wavelets.

The output of CWT was using Python functions of pywt (ver. 1.1) and Matplotlib (ver. 3.3): *x*-axis representing time, *y*-axis representing scale (analogous to frequency), and *z*-axis showing coefficient value. Then, a scalogram is plotted as a smooth 2D image of time and frequency, and the amplitude of the frequency components is shown by varying the color or intensity of that point. Typically, dark blue colors represent the low amplitudes and bright yellow colors mean large amplitude coefficients. The PPG signal is transformed into different scalograms using Frequency B-Spline wavelet (fbsp1-15-1), Shannon wavelet (shan15-1), Complex Gaussian wavelet (cgau1), Morlet wavelet (morl), Mexican hat wavelet (mexh), and Gaussian wavelet (gaus1) for testing. The transformed colorful scalograms by different wavelets are seen in Figure 1. The list included the equations and parameters of these analytic wavelets:

(1) $$\begin{array}{c} \text{fbsp}:\text{ fbsp}\left(t\right)=\sqrt{F_{b}}+{\left(\sin\;c\left(\frac{F_{b}t}{m}\right)\right)}^{m}e^{2i\pi F_{c}t},\;\left(m=1,\;F_{b}=15,\;F_{c}=1\right). \end{array}$$

Parameter explanation: *m* is an integer order parameter (≥1); *F*_*b*_ is bandwidth; and *F*_*c*_ is a wavelet center frequency:

(2) $$\begin{array}{c} \text{shan}:\text{ shan}\left(t\right)=F_{b}{}^{0.5}\sin\;c\left(F_{b}t\right)e^{2i\pi F_{c}t},\;\left(F_{b}=15,\;F_{c}=1\right). \end{array}$$

Parameter explanation: *F*_*b*_ is bandwidth; *F*_*c*_ is a wavelet center frequency; and the condition *F*_*c*_ > *F*_*b*_/2 is sufficient to ensure that zero is not in the frequency support interval:

(3) $$\begin{array}{c} \text{cgau}1:\text{ cgau}\left(t\right)=C_{n}\text{diff}\left(e^{-it}e^{-x^{2}},n\right)\;\left(n=1\right). \end{array}$$

Parameter explanation: the number “*n*” means vanishing moments, where diff denotes the symbolic derivative and *C*_*n*_ is a constant:

(4) $$\begin{array}{c} \text{morl}:\text{ morl}\left(t\right)=\;e^{-x^{2}/2}\cos\left(5t\right),\\ \text{mexh}:\text{ mexh}\left(t\right)=\;\frac{2}{\sqrt{3}\pi^{1/4}}e^{-t^{2}/2}\left(1-t^{2}\right),\\ \text{gaus}1:\text{ gaus}\left(t\right)=C_{n}\text{diff}\left(e^{-x^{2}},n\right),\;\left(n=1\right). \end{array}$$

Parameter explanation: the number “*n*” means vanishing moments, where diff denotes the symbolic derivative and *C*_*n*_ is such that the 2-norm of gaus (*x*, *n*) = 1.

### 2.5. Segment Length

The recordings divided into different segments by different units containing reference BPs for analysis are listed here, and all the images were converted to 224 *∗* 224 *∗* 3-pixel size:

  0.8 s (segment 100): train 2799, validation 311.
  1.2 s (segment 150): train 1866, validation 207.
  1.6 s (segment 200): train 1399, validation 155.
  2.0 s (segment-250): train 1118, validation 124.
  2.4 s (segment-300): train 931, validation 103.
  2.8 s (segment 350): train 798, validation 88.
  3.2 s (segment 400): train 698, validation 77.
  3.6 s (segment-450): train 620, validation 69.
  4.0 s (segment 500): train 558, validation 62.

The accuracy of each CWT using different segments for BP classification was illustrated as a line chart to compare which length of the segment was better.

### 2.6. Convolutional Neural Networks

**Figure 2:**
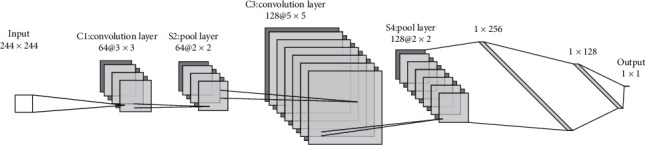
The layers of the proposed convolutional neural networks.

Our proposed CNN is a deep learning tool applied to analyzing visual imagery (Figure 2). A classical CNN model consists of input and output layers, as well as several hidden layers [15]. The input is the CWT generated from the PPG signal that was sorted as 224 *∗* 224 *∗* 3-pixel image. Scalogram images transformed from MATLAB (Morse wavelet) were also tested on our CNN. The hidden layers including two convolution layers (C1 and C3), two pool layers (S2 and S4), and two fully connected layers (F5 and F6) define the core architecture of the network, where most of the computation and learning take place. The F7 (output layer) containing 1 neuron (sigmoid activation) carries on the work into a logistic function using sigmoid.

  C1: 64 kernels of size (3 *∗* 3) with a stride setting of one and the same padding (ReLU activation).
  S2: max pooling (pool size is 2) with a stride of two and the same padding.
  C3: 128 kernels of size (5 *∗* 5) with a stride of one and the same padding (ReLU activation).
  S4: max pooling (pool size is 2) with a stride of two and the same padding.
  F5: 256 neurons (ReLU activation).
  F6: 128 neurons (ReLU activation).

We also performed the transfer learning through pretrained CNN-GoogLeNet which can be directly applied to other computer vision identification. The transformed signals by CWT were also converted to 224 *∗* 224 *∗* 3 sized images and then ran on the CNN-GoogLeNet. It is aimed at comparing our work to the previous study using MATLAB Morse wavelet transformed scalogram [13] and CNN-GoogLeNet.

### 2.7. Accuracy Evaluation

All the accuracy of different CWTs and segments were calculated and shown on the line chart. The accuracy of the best three combinations testing on our proposed CNN was displayed using receiver-operating characteristic (ROC) curve analysis. Then, we applied our best match of CWT and segment using our proposed CNN and transfer learning on CNN-GoogLeNet [16] to compare with MATLAB scalogram. The accuracy comparison and overfitting evaluation were present through loss and accuracy curves.

## 3. Results

### 3.1. Data Overview

Normal BP: 1641 samples; elevated BP: 1739 samples; Stage I hypertension (I-HT): 2692 samples; Stage II hypertension (II-HT): 334 samples;

normal BP: 104.81 ± 10.49/71.55 ± 3.94; abnormal BP: 132.22 ± 4.76/85.09 ± 6.92.

### 3.2. Comparison of Overall Accuracy

**Figure 3:**
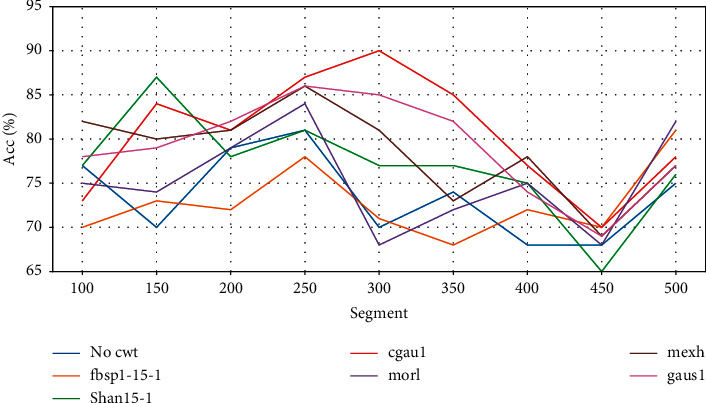
The accuracies of different algorithms by matching CWTs with different segment lengths.

Table 1 shows all the testing accuracy of different CWTs and segments. Most of the accuracy was more than 70%; the lowest accuracy was 65%. The cgau1 with 2.4 s segment revealed the best performance with accuracy of 91%.  Figure 3 shows the accuracy of different CWTs fluctuated up and down with different segments. The peaks of CWTs mostly appeared at the segments in the range of 250 (2.0 s) ∼ 300 (2.8 s).

### 3.3. Accuracy Evaluation from ROC

**Figure 4:**
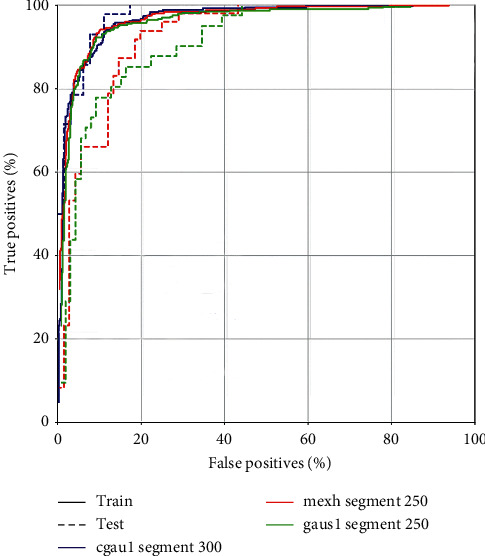
The receiver-operating characteristic curves of cgau1 and segment-300; gaus1 and segment-250; and mexh and segment-250 for prediction of blood pressure classification.

The first three best combinations of CWT and segment were cgau1 and segment-300; gaus1 and segment-250; and mexh and segment-250. The accuracies were 90%, 86%, and 86%, respectively. The accuracy based on the testing set was close to the accuracy of the training set in cgau1, so there was no overfitting problem from the ROC (Figure 4).

### 3.4. The Cgau1 and Segment-300 Examples of Different BP Categories

**Figure 5:**
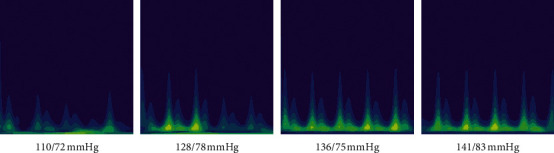
The image examples transformed from cgau1 for different blood pressure categories.

The image examples of different BP categories transformed from cgau1 are shown in Figure 5. The images transformed from cgau1 and segment-300 can be distinguished visually by the naked eyes. The feature of the normal BP (110/72 mmHg) image is smaller peaks and bright yellow at the dexter base. The feature of elevated BP (128/78 mmHg) image is bright yellow at the center bottom of the left higher peaks. The features of I-HT and II-HT are similar with minor variations.

### 3.5. Comparison with Previous Work

**Figure 6:**
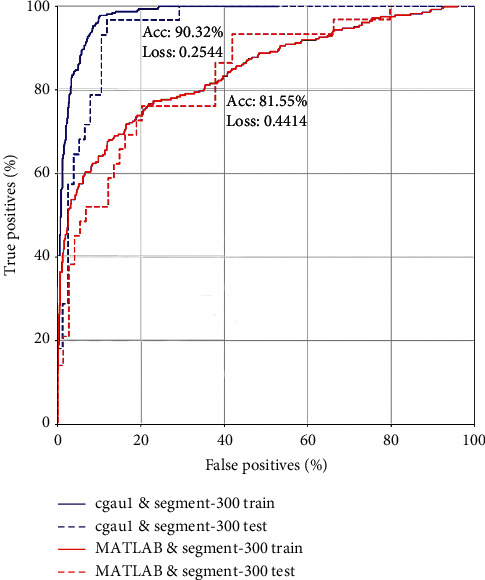
The accuracy of cgau1 and segment-300 and MATLAB scalogram and segment-300 in our proposed CNN from the receiver-operating characteristic curves.

We compared cgau1 and segment-300 to scalogram and segment-300 transformation previously used by other studies. The ROC (Figure 6) showed that the accuracy of cgau1 and segment-300 (90%) was better than that of MATLAB Morse and segment-300 (82%) in our proposed CNN.

**Figure 7:**
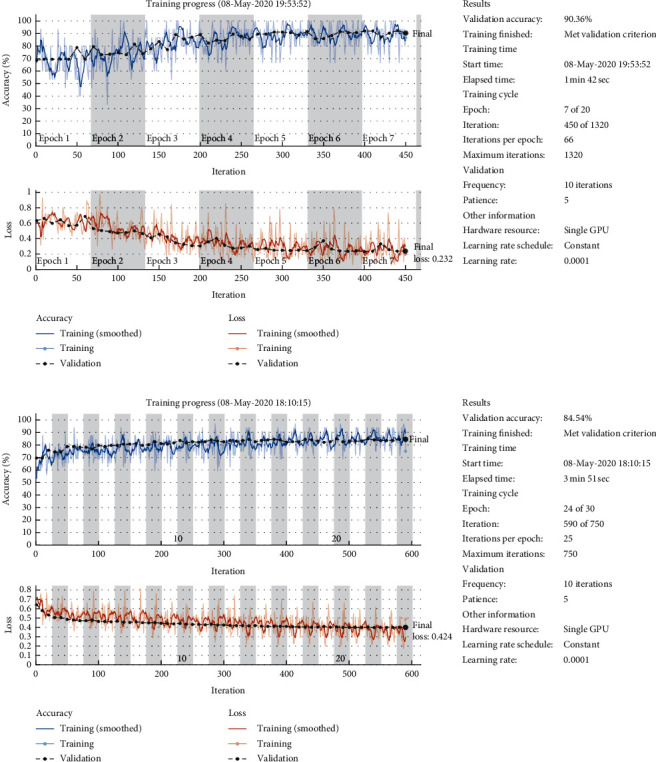
The loss and accuracy training process of cgau1 and segment-300 and MATLAB scalogram and segment-300 in CNN-GoogLeNet by transfer learning.

When the cgau1 and segment-300 and MATLAB Morse and segment-300 methods were tested on CNN-GoogLeNet by transfer learning, cgau1 and segment-300 also performed better than MATLAB Morse and segment-300. The loss and accuracy training process (Figure 7) showed that the accuracy and loss were 90.36% versus 84.5% and 0.23 versus 0.42, respectively. There was also no overfitting in both. It means that our best model was efficient in other CNN models and stable for predictions when new data were applied.

## 4. Discussion

PPG is a low-cost, miniature, and wearable optical biosensor that can be applied to cardiovascular monitoring, including the detection of blood oxygen saturation, heart rate, BP, and cardiac output [17, 18]. Waveform propagation and waveform morphology are the two main methods to detect BP from PPG [19]. The waveform propagation method requires multiple sensors, and the issue of synchronization of signals increases the difficulty and instability of BP analysis. Documented PPG and ECG signal data from MIMIC are not perfectly synchronized. Since the PPG signal produces pulse waveforms that are very similar to pressure waveforms generated by tonometry, there is clear evidence that the BP fluctuations are reflected in the PPG waveform morphology [20]. The PPG signal is a complex mixture with a coverage area of arteries, veins, and numerous capillaries [21]. A raw PPG signal provides much information about the circulatory system generated from pulsatile and nonpulsatile blood volume [22]. Then, it is not easy to extract the features of BP information from raw messy signals by waveform morphology. Slope transit time (STT) [23] and BD area [24] were correlated with BP but are far from the accurate prediction of BP. The CWT is an effective tool to expose the characteristics of different BP levels transformed from raw PPG signals. Figure 7 in our study clearly illustrated the features of different BP categories, and the transformed images are easy to recognize. Deep learning technology plays a pivotal role in image recognition [25] and is qualified for the work of classifying the CWT scalograms. The results in our study showed that except for fbsp1-15-1, the accuracy of CWTs was higher than that of no CWT when testing on our proposed CNN. CNN use relatively little preprocessing compared to other image classification algorithms, which has gained a lot of popularity in this field [26]. Liang et al. uses the MATLAB scalogram and pretrained CNN (GoogLeNet) to classify BP, and the three classification trials of normotension versus prehypertension, normotension versus hypertension, and normotension + prehypertension versus hypertension F1-scores were 80.52%, 92.55%, and 82.95%, respectively [13]. The accuracy of the MATLAB scalogram on our proposed CNN was 81.55%, which was similar on CNN-GoogLeNet. Cgau1 transform performed better compared with MATLAB default scalogram on both of our proposed CNN and CNN-GoogLeNet. These results indicated that different CWTs would affect the accuracy of BP classification via deep learning.

Few studies reported whether the segment length could influence the accuracy of BP estimation via PPG. Most studies extracted a 5-second segment as a testing unit [13, 27]. The longer the segment is, the smaller the sample size it will make. The MIMIC dataset is finite, and the 5-second segment will reduce the sample size for testing and validation, so we used the segment units under 4 seconds to keep enough sample size for testing. Interestingly, the performance was quite dissimilar using different segment units. Most of the CWTs performed best on segment-250 (2.0 s) and segment-300 (2.4 s). The low accuracy appeared at segment-450 in all the CWTs. It tells us that a shorter length of the segment would lower the accuracy for prediction, but it does not mean the longer the better.

The combination of appropriate CWT, segment length, and deep learning model will construct the optimal algorithm for accurate prediction. The combination of cgau1 CWT and segment-300 (2.4 s) in our work is the best solution using CNN for BP classification. The method performed better than MATLAB scalogram and segment-300 [13]. The accuracies are quite similar when tested on self-established CNN and CNN-GoogLeNet of transfer learning. Thus, our model is a feasible and promising solution for continuous cuffless BP monitoring on different platforms. If this low-cost, cuffless BP monitors can be developed, it is likely that conventional paradigms of BP measurement would be disrupted. Even some special medical conditions can also benefit from this device; for example, it is proven that cuffless BP measurement still shows high accuracy and reliability in arrhythmia patients [28] and children [29]. Although the PPG signal is altered in individuals suffering from obesity [30], it can be optimized based on multiple types of information fusion [31].

There are some limitations to our study. Firstly, we did not test all the wavelets on the MIMIC dataset, and cgau1 may be not the best CWT for BP classification. Other wavelets, such as Gabor [32] and Paul [33] are the potential to exceed the ability of cgau1 and segment-300 to predict BP estimation. Secondly, our model is not validated on new data and whether the accuracy will keep as high as 90% in real-life scenarios is still unknown. Thirdly, there is a “length effect” on the accuracy of BP assessment by CWT signals, as 2.0 s and 2.4 s were the optimal segments, but a longer segment unit did not improve the accuracy. The possible reason is that longer segments could reduce the amount of training data, which may influence the training result for deep learning. We need more data to confirm the length effect.

## 5. Conclusion

Type of CWT and segment length are the determinants for improving the accuracy to forecast blood pressure classification from PPG using deep learning. The cgau1 and segment-300 method performed better than the previously established MATLAB Morse scalogram and segment-300 approach on both of our proposed CNN and CNN-GoogLeNet. Thus, we established a new algorithm with high accuracy to predict blood pressure classification from PPG via matching of CWT type and segment length. Our study provides a promising solution that can be applied to the real-time processing of the PPG signal from the wearable device for rapid classification of BP.

## Figures and Tables

**Table 1:** All the testing accuracy of different CWTs and segments to predict classification of blood pressure.

Accuracy (%)	100 (0.8 s)	150 (1.2 s)	200 (1.6 s)	250 (2.0 s)	300 (2.4 s)	350 (2.8 s)	400 (3.2 s)	450 (3.6 s)	500 (4.0 s)
No CWT	77	70	79	81	70	74	68	68	75
fbsp1-15-1	70	73	72	78	71	68	72	70	81
shan15-1	77	84	78	81	77	77	75	65	76
cgau1	73	84	81	87	90	85	77	70	78
morl	75	74	79	84	68	72	75	68	82
mexh	82	80	81	86	81	73	78	69	77
gaus1	78	79	82	86	85	82	74	69	77

## Data Availability

All the data generated and analyzed during this study are included in this article.  The supplementary materials have been uploaded on https://github.com/hao203/journal-file/tree/main/ppg-bp.

## Abbreviations

ABPM: Ambulatory blood pressure monitoring
ACC/AHA: American College of Cardiology/American Heart Association
BP: Blood pressure
CNN: Convolutional neural network
CVD: Cardiovascular disease
CWT: Continuous wavelet transform
ECG: Electrocardiogram
FFT: Fast Fourier transform
HBPM: Home blood pressure monitoring
ICU: Intensive care unit
MIMIC- III: Multiparameter Intelligent Monitoring in Intensive Care-III
PAT: Pulse arrival time
PPG: Photoplethysmography
PTT: Pulse transit time.

## Supplementary Materials

Supplementary Materials S_file 1: Python code for scalograms transformed from PPG by continuous wavelet transform. S_file 2: Python code for predicting classification of blood pressure by deep learning. S_file 3: the dataset from Waveform Database provided by PhysioNet.
